# AIMP2-DX2 Promotes the Proliferation, Migration, and Invasion of Nasopharyngeal Carcinoma Cells

**DOI:** 10.1155/2018/9253036

**Published:** 2018-04-16

**Authors:** Qingsong Cao, Jie Zhang, Tao Zhang

**Affiliations:** ^1^Department of Otorhinolaryngology, The First Affiliated Hospital, Jinan University, Guangzhou 510630, China; ^2^Department of First Class Ward, The First Affiliated Hospital, Jinan University, Guangzhou 510630, China

## Abstract

Nasopharyngeal carcinoma (NPC) is a head and neck tumor with high degree of malignancy and with high incidence especially in southern China. AIMP2-DX2, one isoform of the aminoacyl-tRNA synthetase interacting multifunctional proteins (AIMPs), is shown to be a potential target in many cancers. However, the detailed mechanisms of AIMP2-DX2 in NPC development remain to be elucidated. Here, we found that the mRNA expression level of AIMP2-DX2 was significantly increased in NPC specimens, compared with normal nasopharyngeal tissues. Microarray immunohistochemical analysis of NPC specimens and Kaplan–Meier analysis showed that patients with high AIMP2-DX2 protein expression had shorter overall survival than those with low AIMP2-DX2 level. Furthermore, mRNA and protein expression levels of AIMP2-DX2 were both increased in cultured NPC cell lines (5-8F, CNE-2Z, and CNE-1), by being compared with normal nasopharyngeal cell line NP69. Overexpression of AIMP2-DX2 remarkably promoted the cell viability, cell migration, and invasion of cultured NPC cells. Genetic knockdown of AIMP2-DX2 by shRNA lentiviruses significantly suppressed the proliferation, migration, and invasion and induced apoptosis of NPC cells. Inhibition of AIMP2-DX2 decreased the highly expressed level of matrix metalloproteinase- (MMP-) 2 and MMP-9, further suppressed proliferation, migration, and invasion in cultured NPC cells* in vitro*, and inhibited tumor growth in a xenograft mouse model* in vivo.* Taken together, these results suggest that AIMP2-DX2 plays an important role in the regulation of NPC and could be a potential therapeutic target and prognostic indicator for the treatment of NPC.

## 1. Introduction

Nasopharyngeal carcinoma (NPC) is a rare tumor derived from nasopharyngeal epithelium [1], most of which are poorly differentiated squamous cell carcinomas [2]. NPC is prevalent in east Africa and Asia, especially southern China, with the incidence rate of up to 0.2% [3, 4]. According to epidemiological studies, the occurrence of NPC may be closely related to genetic factors, environmental factors, and Epstein–Barr Virus (EBV) infection [5, 6]. People of smoke, alcohol abuse, and exposure to dangerous chemicals show higher risk of NPC [7]. Until now, the treatment of NPC still remains as the greatest obstacle, due to the high rate of metastasis [8, 9]. Thus, elucidating the mechanisms and screening biomarkers for early diagnosis show great clinical significance.

**Figure 1:**
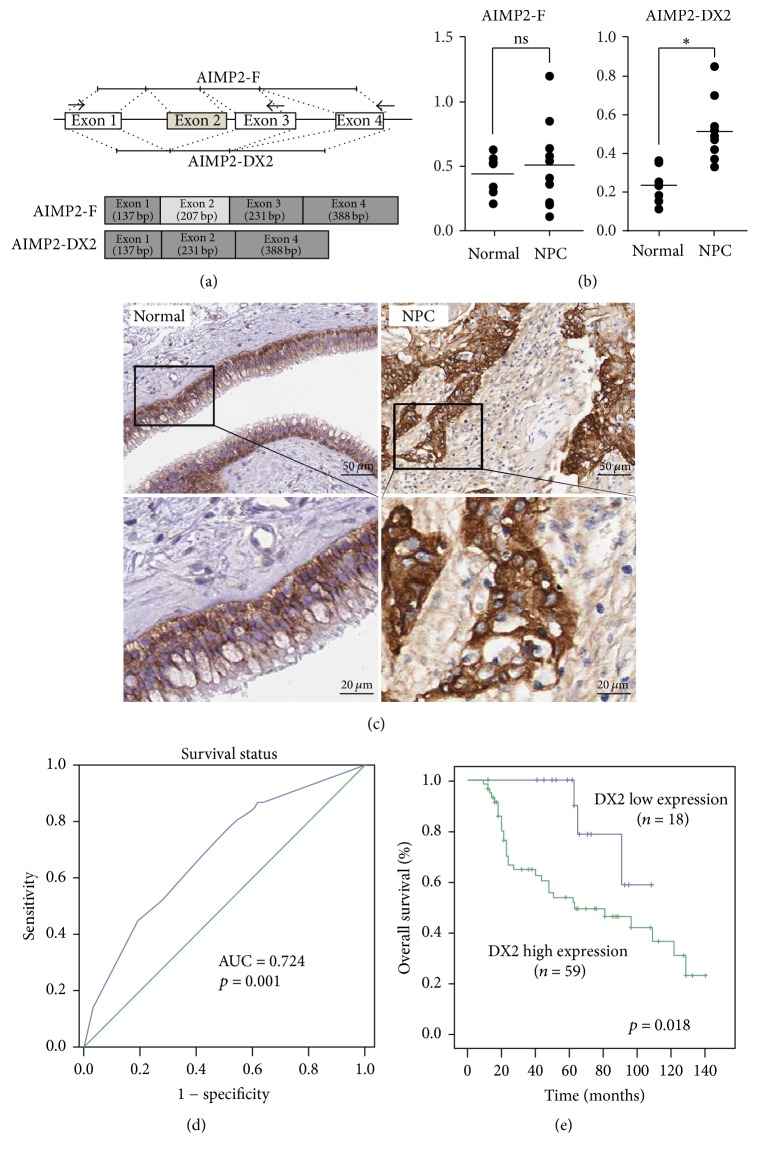
*AIMP2-DX2 is upregulated in NPC specimens and correlated with poor diagnosis.* (a) Schematic diagram of AIMP2 and AIMP2-DX2 transcripts (AIMP2-F, full-length AIMP2) [16]; (b) qPCR detecting the mRNA level change of AIMP2 and AIMP2-DX2 in NPC patients. Normal: normal nasopharyngeal tissue; NPC: nasopharyngeal carcinoma tissue. *∗* denotes *p* < 0.05; ns indicates no difference. (c) Immunohistochemical staining of AIMP2-DX2 expression in normal nasopharyngeal tissues and in NPC specimens. Enlarged local images were shown in below panel. ROC curve (d) and Kaplan–Meier survival curves (e) showed the relationship between expression of AIMP2-DX2 and survival.

AIMP2 (Aminoacyl-tRNA synthetase interacting multifunctional protein 2, also known as p38/JTV-1), one isoform of the three AIMPs, is involved in cancer development. AIMP2 functions in a variety of biological processes, such as cell differentiation and apoptosis [10, 11]. AIMP2 induces cell apoptosis via activation of p53 and downregulation of TRAF2 [12, 13], or via regulation of c-Myc expression level [14, 15]. AIMP2-DX2 is another isoform of AIMP2, but it lacks the Exon 2 (Figure 1(a)) [16]. Recent studies show that AIMP2-DX2 is also involved in cancer development. AIMP2-DX2 is closely correlated to the severity of lung cancer and is positively related to drug resistance in ovarian cancer [16, 17]. Inhibition of AIMP2-DX2 may suppress the proliferation of lung cancer cells and restrain the tumor formation in nude mice [18, 19]. Thus AIMP2-DX2 acts as an oncogene, different from AIMP2.

However, the role of AIMP2-DX2 in NPC remains unknown. Here, in the current study, we detected the expression level of AIMP2-DX2 in NPC specimens and analyzed its correlation with overall survival. We compared the mRNA and protein expression levels of AIMP2-DX2 in cultured NPC cell lines with normal nasopharyngeal epithelial cells. AIMP2-DX2 was overexpressed and silenced to determine the impact on proliferation, migration, and invasion in NPC cells. Further, we determined the effect of AIMP2-DX2 inhibition on tumor formation in nude mice.

## 2. Materials and Methods

### 2.1. Ethics Statement

This study was approved by the Medical Ethical Committee for Clinical Research and Animal Trials of the First Affiliated Hospital of Jinan University, in accordance with the Declaration of Helsinki. Clinical specimens were collected under the informed consent from all participants. Of animal experiments, all efforts were made to minimize suffering.

### 2.2. Human NPC Specimens

NPC samples were obtained from 77 patients (41 males and 36 females, average age: 48.5  ±  2.2) between January 2008 and December 2012. Normal nasopharyngeal tissues (from nasal polyp) were obtained from 56 cases (average age: 49.6 ± 3.2). The NPC specimens were confirmed as nasopharyngeal squamous cell carcinomas by the Department of Pathology. Samples were fixed with 10% formaldehyde when obtained and then paraffin-embedded.

### 2.3. Immunohistochemistry

The rabbit anti-AIMP2-DX2 antibody was prepared as described [20]. Immunohistochemistry was performed according to our experience. Briefly, samples were deparaffinized and rehydrated; antigen retrieval also was achieved in a microwave in 10 mM citrate buffer at pH 6.0. To inactivate endogenous peroxidases, samples were immersed with hydrogen peroxide 37°C for 30 minutes. Sections were fixed with paraformaldehyde followed by permeabilization and blocking with appropriate preimmune serum. After incubation of anti-AIMP2-DX2 (1 : 200) antibody overnight at 4°C, samples were washed and a HRP-labeled secondary antibody was incubated at 37°C for 1 h. Expression in normal tissue was used as negative control.

### 2.4. shRNA Stable Cell Line Construction

Immortalized nasopharyngeal epithelial cell line (NP69) and NPC cell lines (5-8F, CNE-1, and CNE-2Z) were achieved from Cell Bank of Shanghai Institute of Cell Biology, Chinese Academy of Sciences (Shanghai, China) and cultured in accordance with the protocol. Vectors of pGag/Pol, pRev, and pLL3.7 (Addgene, Cambridge, MA, USA) were used for the construction of recombinant shAIMP2-DX2 lentiviral plasmid. The sequence designed for shRNA construction was 5′-AGAAATTCTCGAATGTTCTTT-3′. HEK 293T cells were cotransfected for virus packaging. 48 and 72 h after transfection, the supernatant was collected for virus enrichment. Viruses of shAIMP2-DX2 and shControl (negative control) were infected in CNE-1 cells, with 5–10 *μ*g/ml polybrene; 48 h later, 2 *μ*g/ml puromycin was added to the culture medium for selection, to generate NPC shRNA stable cell lines.

### 2.5. Western Blot Analysis

Cell lysates were separated in sodium dodecyl sulfate-polyacrylamide (SDS-PAGE) gel electrophoresis and transferred to a nitrocellulose membrane. Western blot membranes were probed with primary antibodies against full-length AIMP2-DX2 (AIMP2-F) and AIMP2-DX2, matrix metalloproteinase (MMP)-2, MMP-9, and Actin (Abcam, MA, USA). Horseradish peroxidase-conjugated secondary antibodies (Amersham Biosciences, Uppsala, Sweden) were incubated for 1 h, and bands were detected by enhanced chemiluminescence (Amersham, Bucks, UK). Densitometric values were normalized to Actin levels, by the analysis of ImageJ software.

### 2.6. Reverse Transcription-Polymerase Chain Reaction (RT-PCR)

Total RNA was extracted with Trizol reagent (Invitrogen, Carlsbad, CA, USA) according to the manufacturer's instruction. cDNAs were extracted and used for amplification of* aimp2-F*,* aimp2-DX2*,* mmp-2*, and* mmp-9*. Primers used were* aimp2-F* (forward, 5′-ACCGGCTCCCCAACGTGCAC-3′),* aimp2-F* (reverse, 5′-AAGTGAATCCCAGCT-3′),* aimp2-DX2* (forward, 5′-ATGCCGATGTACCAGGTAAAGCCCTATC-3′), and* aimp2-F* (reverse, 5′-CTTAAGGAGCTTGAGGGCCGTGTTAAAAG-3′). The statistical results were normalized to the expression of* Actin*.

### 2.7. Cell Viability Assay

Cell proliferation was detected by the MTS assay (Promega, Madison, WI, USA) as reported in [21]. Briefly, cell cultures in 96-well plate were incubated at 100 *μ*l medium with 20 *μ*l of CellTiter 96 AQueous One Solution reagent for 3-4 h. The cell viability was determined. The growth rate was calculated from the absorbance and was normalized.

### 2.8. In Vitro Migration and Invasion Assays

In vitro cell migration and invasion assays were examined by transwell system (Millipore, MA, USA). Briefly, cells were seeded on a polycarbonate membrane insert incubated for 24 h. Cells migrated into the lower surface were stained with 2% crystal violet for quantification. For the invasion assay, the procedure was similar except that the transwell membranes were precoated with Matrigel (Becton Dickinson Bioscience, MA, USA).

### 2.9. Apoptosis by Flow Cytometry

Cells plated in six-well plate were washed twice with PBS and fixed in 70% ethanol at 4°C overnight. Then, cells were stained with propidium iodide and Annexin and analyzed by flow cytometry using a FACScan flow cytometer (BD Biosciences, Mountain View, CA, USA).

### 2.10. In Vivo Tumor Model

Cells (2 × 10^6^) of the shAIMP2-DX2 and shControl stable CNE-1 lines were injected subcutaneously into the flanks of 4-week-old nude mice (*n* = 5 for each group, resp.). Tumor formation in nude mice was administrated for over 4 weeks. Tumor volumes were measured during the period and calculated using the formula *V* = (4/3)*πa*^2^*b* [22]. Following sacrifice after 4 weeks, tumor weight was recorded.

### 2.11. Statistical Analysis

The data are presented as the mean ± SD and statistical analysis was performed by SPSS 20.0 software, using Student's *t*-tests or one-way ANOVAs. To define the cutoff score for high expression of AIMP2-DX2, ROC curve analysis was used. Survival curve was analyzed by Kaplan–Meier method, using the log-rank test. *p* < 0.05 was considered statistically significant.

## 3. Results

### 3.1. AIMP2-DX2 Is Increased in NPC Specimens and Correlated with Poor Prognosis

AIMP2-DX2 has been shown an oncogene in lung and ovarian cancers; however its role in NPC remains to be elucidated. Thus to determine the clinical significance of AIMP2-DX2 in primary human NPC tissue samples, surgical specimens from 77 NPC patients and 56 normal cases in the First Affiliated Hospital of Jinan University between January 2008 and December 2012 were collected for analysis. Transcriptional expression levels of AIMP2-DX2 and full-length AIMP2 (AIMP2-F) were detected via qPCR assay. The results showed that the expression of AIMP2-DX2 in NPC tissues (*p* < 0.05) was significantly higher than that in normal nasopharyngeal tissues, while that of AIMP2-F showed no significant changes (Figure 1(b)). Further, these specimens were subjected to immunohistochemical microarray for AIMP2-DX2. As shown in Figure 1(c), the staining showed that AIMP2-DX2 mainly located in the cytosol of cancer cells. The correlations between AIMP2-DX2 expression and the clinical characteristics of patients were analyzed. The ROC curves for AIMP2-DX2 showed the point on the curve closet to 0 and 1, which maximized the sensitivity and specificity for overall survival (Figure 1(d)). Clinical 77 NPC cases were divided into two groups: high expression group and low expression group. By Kaplan–Meier method, the 5-year survival rate of AIMP2-DX2 low expression group was significantly higher than that of AIMP2-DX2 high expression group (*p* = 0.018, Figure 1(e)). These data indicate that the increased expression of AIMP2-DX2 is positively correlated with poor clinical outcome of NPC patients.

### 3.2. AIMP2-DX2 Is Increased in NPC Cell Lines

**Figure 2:**
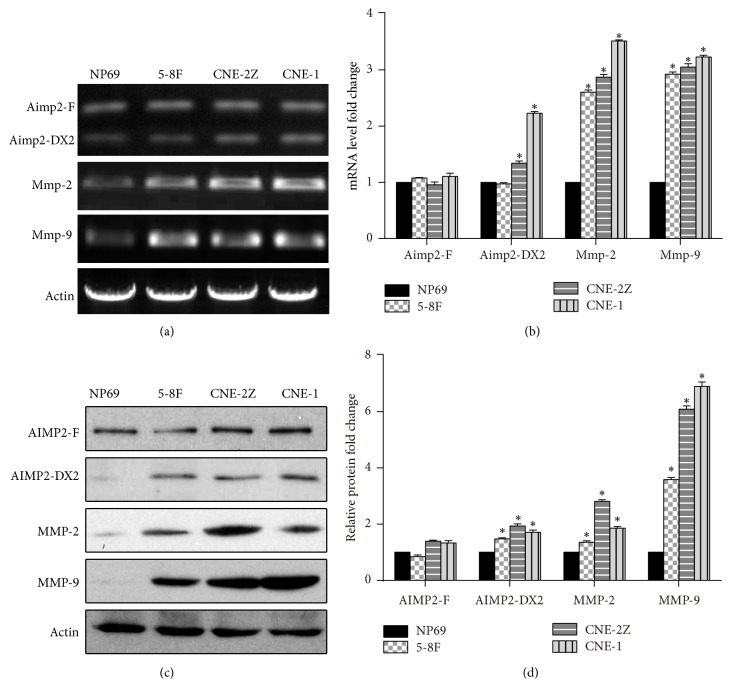
*AIMP2-DX2 is increased in NPC cell lines.* RT-PCR detecting mRNA expression level of AIMP2 and AIMP2-DX2, MMP-2, and MMP-9 in nasopharyngeal cells and nasopharyngeal carcinoma cell lines of human. Representative images were shown in (a) and statistical data were shown in (b). Western blotting detecting mRNA expression level of AIMP2 and AIMP2-DX2, MMP-2, and MMP-9 in nasopharyngeal cells and nasopharyngeal carcinoma cell lines of human. Representative images were shown in (c) and statistical data were shown in (d). *∗* indicates *p* < 0.05, compared with relative expression levels in NP69 cell line.

To further evaluate the role of AIMP2-DX2 in NPC and the downstream mechanisms, the mRNA and protein expression levels were measured by RT-PCR and western blotting in cultured immortalized nasopharyngeal epithelial cell line (NP69) and NPC cell lines (5-8F, CNE-1, and CNE-2Z). AIMP2-DX2 mRNA levels were significantly higher in NPC cell lines than that in the epithelial cell line NP69, while the levels of AIMP2-F showed no changes (Figure 2(a)). Further, we measured the mRNA expression levels of MMP-2 and MMP-9. The results revealed that MMP-2/9 levels were also upregulated in NPC cells (Figure 2(a)). These mRNA levels normalized to Actin expression (loading control) were shown in Figure 2(b). Assessment of these protein levels by western blotting showed a similar trend to that of mRNA levels (Figures 2(c) and 2(d)). These data indicate that AIMP2-DX2 is significantly overexpressed in NPC.

### 3.3. Overexpression of AIMP2-DX2 Promotes the Proliferation, Migration, and Invasion of NPC Cells

**Figure 3:**
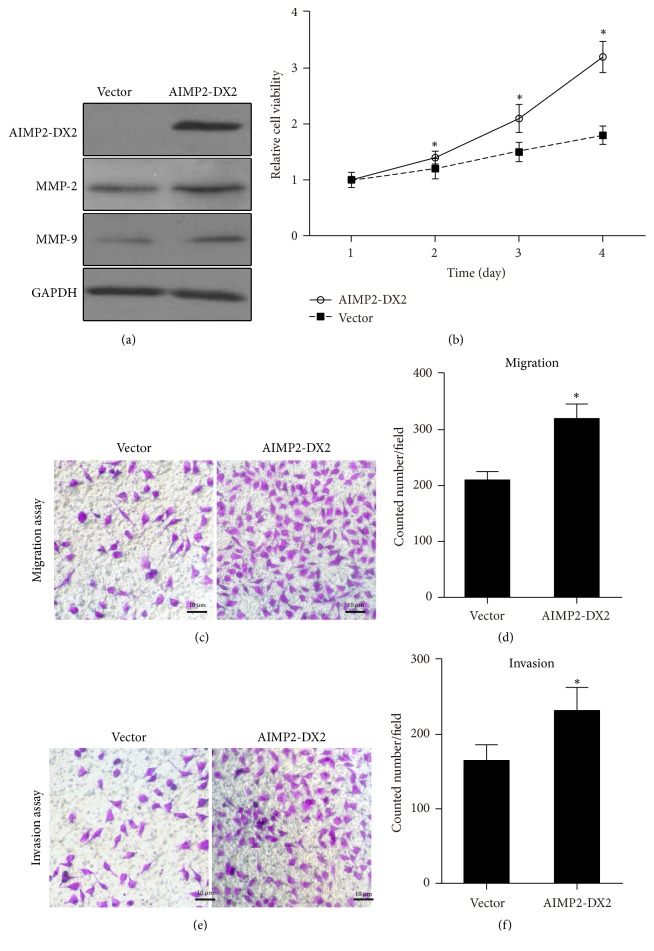
*Overexpression of AIMP2-DX2 promotes the proliferation, migration, and invasion of CNE-1 NPC cells.* AIMP2-DX2 was overexpressed in CNE-1 cells; western blotting detected gene expression of AIMP2-DX2, of GAPDH as control (a). CNE-1 cells of AIMP2-DX2 overexpressed were subjected to MTS assay to evaluate the effect on cell growth (b) and to transwell assay for migration (c, d) and invasion (e, f) assay. *∗* indicates *p* < 0.05, compared with control.

Because of the relatively high expression of AIMP2-DX2, CNE-1 cell line was selected to further verify the impact of AIMP2-DX2 on NPC cell behavior. We first constructed an AIMP2-DX2 overexpression encoding plasmid. The expression efficacy was confirmed by western blotting with His-Tag antibody when overexpressed in CNE-1 cells (Figure 3(a)). Then CNE-1 cells of AIMP2-DX2 overexpressed were subjected to MTS assay for cell proliferation test and to transwell assay for migration and invasion tests. As shown in Figure 3(b), AIMP2-DX2 overexpression significantly increased the cell viability, compared to control vector. Moreover, cells were subjected to transwell assay. As shown in Figures 3(c) and 3(e), AIMP2-DX2 overexpression significantly promoted the migration and invasion ability of CNE-1 cells. Statistical data were shown in Figures 3(d) and 3(f). Moreover, we found that overexpression of AIMP2-DX2 increased the endogenous levels of MMP-2 and MMP-9 (Figure 3(a)), indicating the possible downstream effector of AIMP2-DX2. These data indicate that AIMP2-DX2 is sufficient for the proliferation, migration, and invasion of cultured NPC cells.

### 3.4. Knockdown of AIMP2-DX2 Suppresses the Proliferation, Migration, and Invasion and Induces Apoptosis of NPC Cells

**Figure 4:**
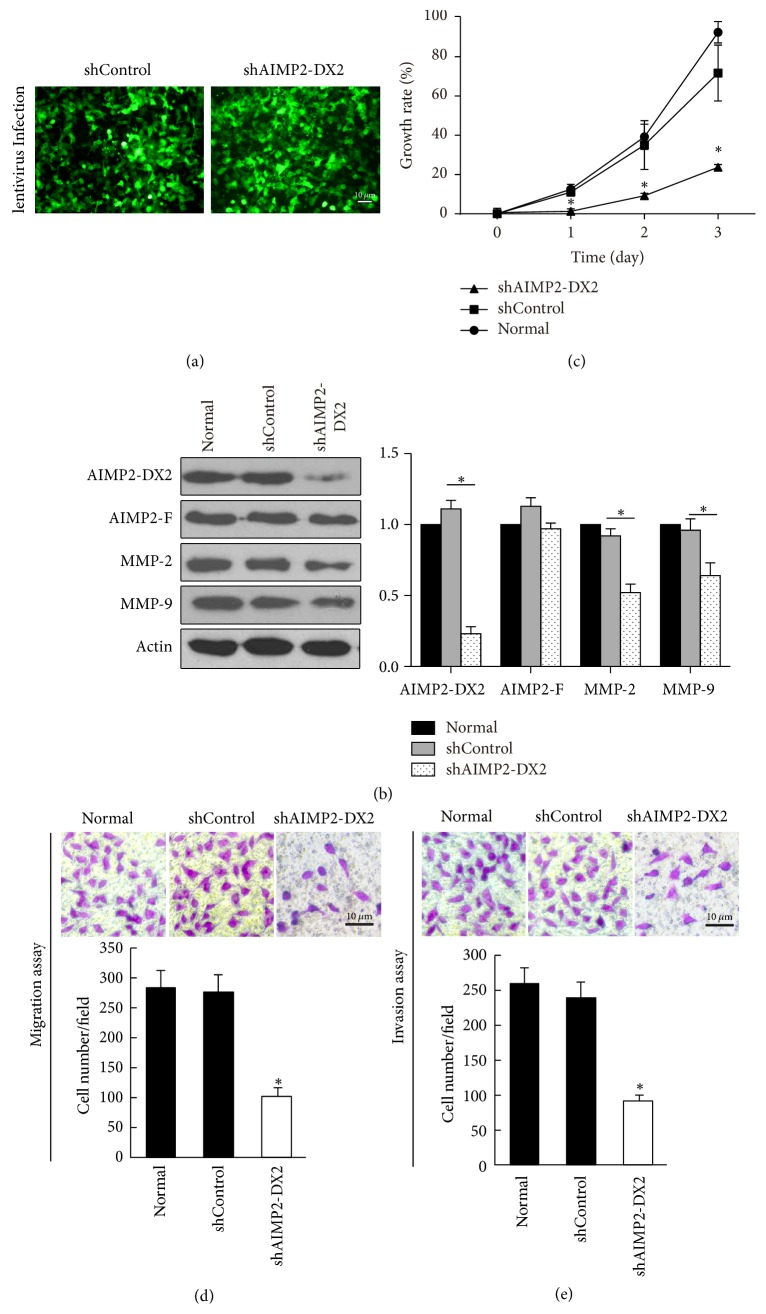
*Impact of silencing AIMP2-DX2 on NPC cell CNE-1.* (a) Representative images after shControl lentivirus and shAIMP2-DX2 lentivirus infection in cell CNE-1. (b) Western blotting detecting protein expressions, (c) MTS assay detecting cell growth and transwell assay detecting cell migration (d) and invasion (e) by AIMP2-DX2 silencing on CNE-1 cell line. *∗* indicates *p* < 0.05, by being compared with shControl group.

**Figure 5:**
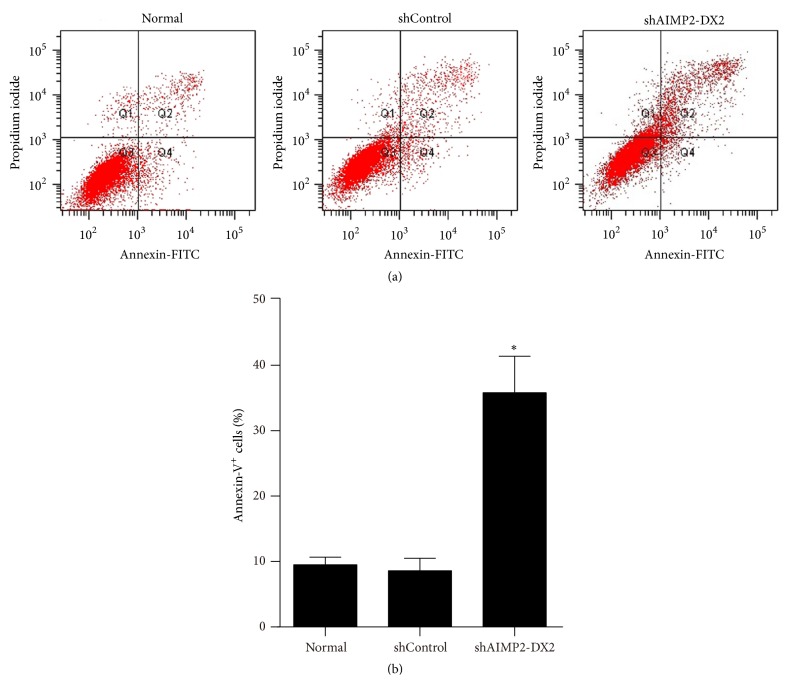
*AIMP2-DX2 silencing induces apoptosis in CNE-1 cells.* Representative images of AnnexinV-FITC/PI double staining of flow cytometry detecting impact of AIMP2-DX2 gene silencing on apoptosis were shown in (a) and statistical data were shown in (b). *∗* indicates *p* < 0.05, by being compared with shControl group.

Further, we wondered about the impact of AIMP2-DX2 inhibition on NPC cells. Genetic silencing sequences against AIMP2-DX2 were designed and synthesized as described in the method section; then an AIMP2-DX2 shRNA lentivirus was constructed (Figure 4(a)), while the scrambled sequence targeting no gene was used as negative control (shControl). Protein expression was applied to determine the shRNA efficacy, by western blotting after lentivirus preparation. As shown in Figure 4(b), the expression level of AIMP2-DX2 was significantly downregulated by the shRNA lentivirus, with AIMP2-F (full-length AIMP2) and Actin expression levels not affected. Interestingly, the expression levels of MMP-2/9 were also remarkably suppressed along with the expression of AIMP2-DX2. Next, cell proliferation was assessed by MTS assay and cell migration by transwell assay. The results showed that knockdown of AIMP2-DX2 inhibited cell growth (Figure 4(c)) and significantly suppressed the migration and invasion of CNE-1 (Figures 4(d) and 4(e)). The impact of AIMP2-DX2 knockdown on cell apoptosis was assessed by flow cytometry. As shown in Figure 5, genetic silencing of AIMP2-DX2 significantly increased the percentage of Annexin-V positive cells, indicating the increased cell apoptosis rate. Taken together, these data suggest that AIMP2-DX2 is necessary for NPC development. AIMP2-DX2 plays a critical role in cell proliferation, migration, and invasion of NPC cell* in vitro*.

### 3.5. AIMP2-DX2 Silencing Inhibits the Growth of NPC Cells In Vivo

**Figure 6:**
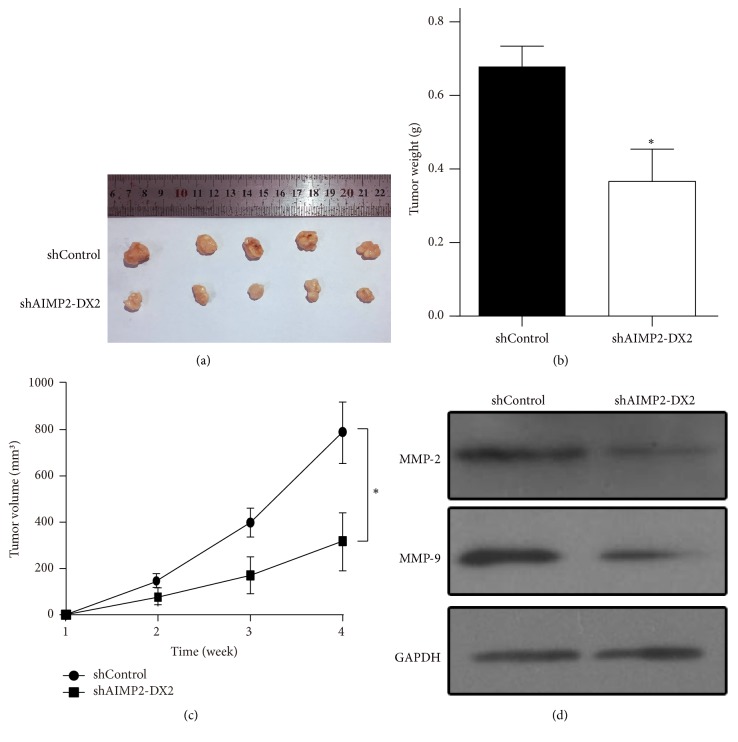
*Inhibition of AIMP2-DX2 suppresses tumor growth in nude mice model.* shAIMP2-DX2 CNE-1 cells were injected into nude mice and were observed for 4 weeks. Then mice were sacrificed, the tumors in shControl and shAIMP2-DX2 groups were shown in (a), and tumor weights were shown in (b); tumor volumes detected from each week were shown in (c). *∗* indicates *p* < 0.05, by being compared with shControl group.

Further, we investigated whether AIMP2-DX2 inhibition would affect NPC cell growth in nude animal model* in vivo*. Cells of shAIMP2-DX2 and shControl CNE-1 stable cell lines were inoculated into the flanks of nude mice. Tumor volumes were assessed every week and mice were sacrificed 4 week later. The data showed that AIMP2-DX2 knockdown significantly suppressed tumor growth, compared with that in control mice (Figure 6(a)). The tumor weight (Figure 6(b)) and tumor volume (Figure 6(c)) were significantly decreased in shAIMP2-DX2 group. Furthermore, we detected the expression levels of MMP-2/9 and found that inhibition of AIMP2-DX2 resulted in decreased expression of MMP proteins (Figure 6(d)). In general, these data suggest that AIMP2-DX2 is involved in NPC development, and genetic knockdown of AIMP2-DX2 suppresses tumor cell growth of NPC* in vitro *and* in vivo*.

## 4. Discussion

NPC is a rare tumor derived from nasopharyngeal epithelia. In China, NPC is a common cancer with a trend of increasing incidences from North to South. Generally, the occurrence of NPC is a process of multifactor, multigene, and multipathway. EB viruses and chemical inducers are believed to be the main risk factors of NPC [5, 6]. During recent years, as the economy is developing, the environment is becoming much more polluted; toxic particles in the air may be another risk factor for NPC [23, 24]. Accordingly, about 70% of NPC patients are diagnosed as the late cancer stage at the first visit, with a strong metastasis trend and poor prognosis. This is the main reason for treatment failure of NPC. Thus, the search for early clinical markers is of significant importance for the treatment of NPC. Here, in the current study, we demonstrate that AIMP2-DX2 is upregulated in NPC specimens and in cultured cells. AIMP2-DX2 is sufficient and necessary for the proliferation, migration, and invasion of NPC cells. Inhibition of AIMP2-DX2 suppresses the growth and metastasis of NPC cells. The data indicate that AIMP2-DX2 is an important oncogene of NPC development, providing new evidence for the treatment of NPC.

AIMP2 is firstly identified as a cofactor of multi-aminoacyl-tRNA synthetase complex (MSC). Recent studies show that AIMP2 can be departed from the complex to inhibit the development of cancers, via interacting with damaged DNA or mediating ubiquitin of substrates [25, 26]. During DNA damage, AIMP2 interacts with p53 to stop its MDM2-dependent ubiquitin pathway [13]. AIMP2 would mediate the ubiquitin of FBP under the stimulus of TGF-*β* [10]. Also, AIMP2 can mediate TNF-*α* induced ubiquitin degradation of TRAPF2 [12]. All this evidence suggests that AIMP2 is a tumor suppressor. AIMP2-DX2, the exon2-lacking isoform of AIMP2, with the same sequences and structure of AIMP2, binds similar interaction proteins of AIMP2. Once the expression ratio of AIMP2-DX2 increased, the function of AIMP2 would be competitively inhibited, acting like an oncogene. Thus, the ratio of AIMP2-DX2/AIMP2 is critical for the development of cancer. Literature shows that AIMP2-DX2 is specifically expressed in a variety of cancers, including lung, breast, liver, stomach, and bone cancers [14, 17–19]. However, the role of AIMP2-DX2 in NPC remains unelucidated. We found that AIMP2-DX2 was highly expressed in NPC tissues; the ratio of AIMP2-DX2/AIMP2 was significantly upregulated and was correlated with the prognosis of NPC patients. We confirmed AIMP2-DX2 as an important inducer for NPC.

Cancer metastasis is a complex multistep process, generally including four steps: reduction of adhesion, degradation of extracellular matrix, invasion of neovascularization, and formation of proliferative lesion in the secondary part [27]. Invasion of the matrix membrane is the critical step, involving many adhesion molecules, enzymes, and cytokines [28]. EMT (epithelial-mesenchymal transition) is the important process for the metastasis of a variety of epithelial tumors [29]. Matrix metalloproteinases (MMPs) play an important role in the process [30]. MMP-2 and -9 have been reported to be related with the metastasis of NPC [31, 32]. Consistently, the mRNA and protein expression levels of MMP-2/9 were upregulated in NPC cells (Figure 2). Overexpression of AIMP2-DX2 resulted in increased expression (Figure 3(a)) and inhibition in significant decrease of MMP-2/9 (Figures 4(b) and 6(d)), consistent with the reduced numbers of NPC cells with migratory and invasive activities. MMPs also are related to cell proliferation, and our data are consistent with previous reports [33–35]. These results indicate that AIMP2-DX2 functions upstream of MMPs to regulate the growth and metastasis of NPC cells.

In conclusion, the present study describes the relevance of AIMP2-DX2 in NPC development. AIMP2-DX2 is significantly upregulated in NPC specimens and cell lines. Inhibition of AIMP2-DX2 suppresses the proliferation of NPC cells* in vitro *and* in vivo*, via the downstream of MMP proteins. High expression of AIMP2-DX2 is associated with poor prognosis in NPC patients. The data suggest that AIMP2-DX2 may be a potential prognostic biomarker for the detection of NPC.
